# Involution of a Large Parotid Hemangioma with Oral Propranolol: An Illustrative Report and Review of the Literature

**DOI:** 10.1155/2012/353812

**Published:** 2012-11-25

**Authors:** Elpis Mantadakis, Emmanouela Tsouvala, Savas Deftereos, Vassilios Danielides, Athanassios Chatzimichael

**Affiliations:** ^1^Department of Pediatrics, Democritus University of Thrace Faculty Medicine, University General Hospital of Alexandroupolis, 68 100 Alexandroupolis, Thrace, Greece; ^2^Department of Radiology and Medical Imaging, Democritus University of Thrace Faculty Medicine, University General Hospital of Alexandroupolis, 68 100 Alexandroupolis, Thrace, Greece; ^3^Department of Otorhinolaryngology, Head and Neck Surgery, Democritus University of Thrace Faculty Medicine, University General Hospital of Alexandroupolis, 68 100 Alexandroupolis, Thrace, Greece

## Abstract

Propranolol has emerged as a new treatment option for infantile hemangiomas. We describe a 20-month-old boy with a large right parotid hemangioma diagnosed at the age of 37 days. Starting at the age of 2.5 months, he received oral propranolol for 6.5 months. Although the mass regressed, it recurred when propranolol was discontinued. He was successfully retreated at the age of 11 months with propranolol for another 5.5 months without further recurrences. Treatment was tolerated well. Our paper and a review of the literature demonstrate that propranolol appears to be safe and effective for symptomatic infantile parotid gland hemangiomas.

## 1. Introduction

Parotid hemangiomas (PHs) are among the most common causes of facial asymmetry in infants [1, 2]. In children, PHs account for >50% of salivary tumors [3, 4]. The majority of them are already present at birth, while 90% appear within the first year of life [4]. PHs are more common in females [1, 4–7]. Cutaneous involvement of the overlying skin is found in >50% of the cases [2]. 

We present an infant boy with a large PH who demonstrated substantial involution of the lesion after administration of oral propranolol and review the relevant literature.

## 2. Case Presentation

**Figure 1:**
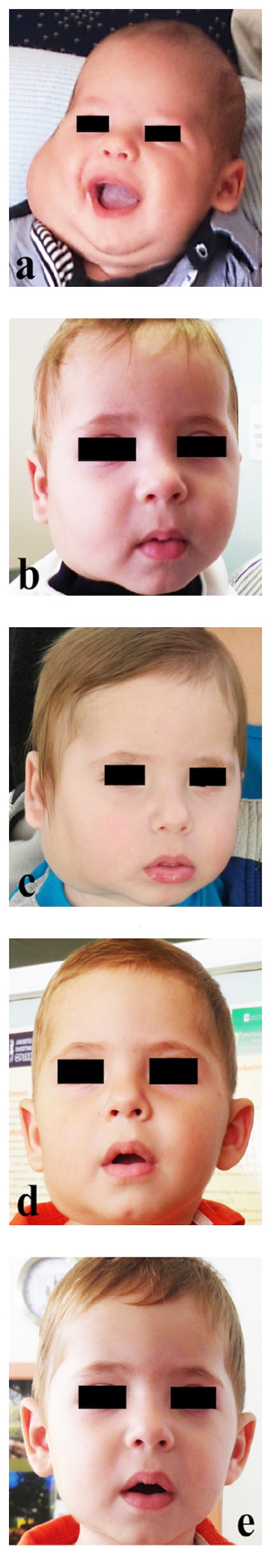
(a) Photograph of the patient at the age of 37 days. A large swelling in the anatomic area of the right parotid gland is obvious. (b) Photograph of the patient at the age of 9 months (end of the first course of propranolol). Marked reduction in the size of the mass is noted. (c) Photograph of the patient at the age of 11 months, that is, just prior retreatment with propranolol was started. There is noticeable regrowth of the mass. (d) Photograph of the patient at the age of 16.5 months (end of therapy). (e) Photograph of the patient 3 months off therapy.

**Figure 2:**
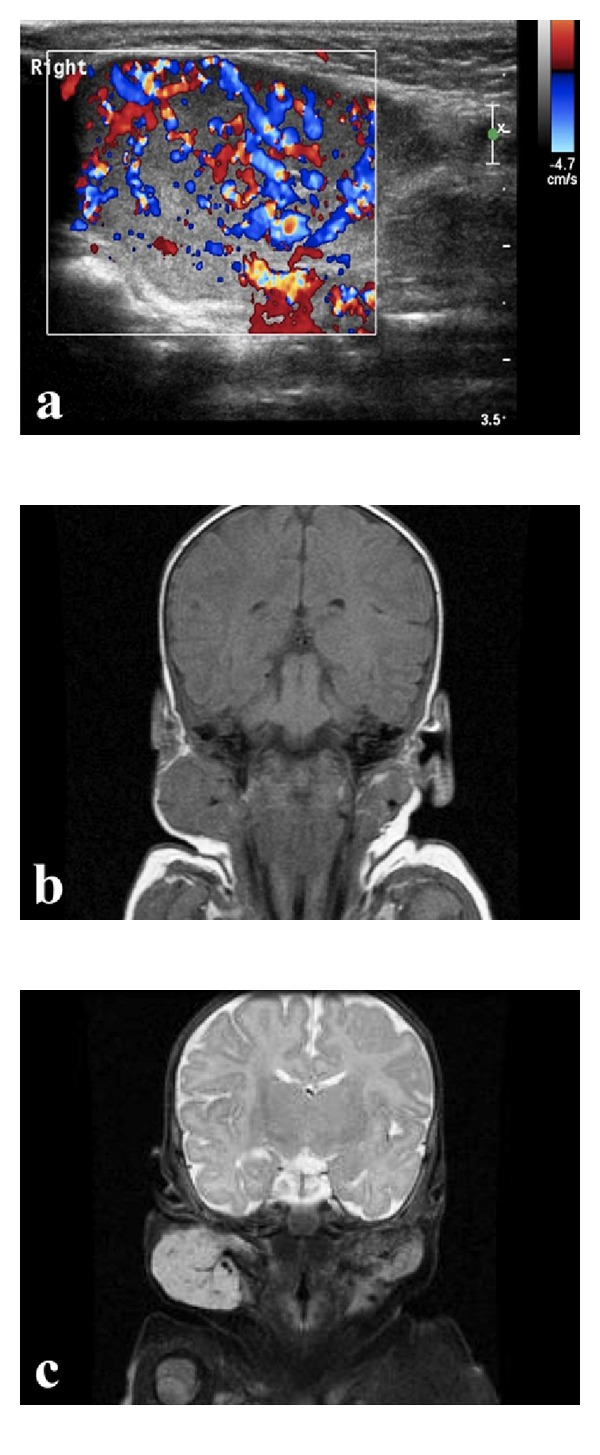
(a) Axial color Doppler sonogram of the right parotid showing an extremely hypervascular gland.(b) Coronal T1-weighted MR image showing a right parotid enlargement that is isointense to muscles. (c) T2-weighted MR image with fat saturation in coronal orientation showing a hyperintense right parotid mass with multiple flow voids.

A 37-day-old male infant presented with acute onset swelling of the right preauricular area (Figure 1(a)). Physical examination showed a soft, painless swelling in the anatomic area of the right parotid gland which was extending beneath the right temporomandibular joint and behind the ipsilateral ear lobe. The overlying skin was normal. An ultrasound disclosed an engorged right parotid gland (4.6 × 2.3 × 3.9 cm) with substantially increased arterial and venous blood flow (Figure 2(a)). MR imaging demonstrated enlargement of the right parotid gland with multiple flow voids, a finding consistent with blood vessels and with low-to-intermediate signal intensity compared to the muscles on short-TR MR images (T1) and bright signal intensity on long-TR images (T2 with fat saturation). No mass effect to middle-line structures of the neck was observed, despite the large size of the lesion (Figures 2(b) and 2(c)). A followup examination 5 weeks later showed further worsening of the facial asymmetry. At this point treatment with oral propranolol 2 mg/kg/day in 3 doses was started. Prior to the administration of the first dose an electrocardiogram was obtained which did not show any AV blocks. During a 2-day hospitalization, the infant did not develop any adverse effects from the treatment. Prior to discharge noticeable softening of the mass was already apparent. He continued treatment for approximately 6.5 months. When the treatment was discontinued, there was only minimal facial asymmetry (Figure 1(b)). However, within days after discontinuation of propranolol, the mass started to regrow. At the age of 11 months (Figure 1(c)), the child was retreated with the same medication and at the same per kilogram dose for another 5.5 months. When the treatment was finally discontinued at the age of 16.5 months, there was a barely noticeable leftover facial asymmetry (Figure 1(d)). The child remains off therapy for >3 months, and he continues to do well (Figure 1(e)). Both times he tolerated propranolol well with no apparent side effects.

## 3. Discussion

Infantile hemangioma (IH) is the most common tumor of the parotid gland in infancy and displays a predilection for females [1]. In a study of 56 children with IH of the parotid gland, 70% were females [2]. Bluish or purple discoloration of the overlying skin is a helpful clinical sign if present, unlike our case [7, 8]. Although it is frequently noticeable at birth, IH typically grows fast and becomes clinically obvious during the subsequent 6 months [3]. In our case the growth was prominent after the 37 day of life. 

Most IHs complete their proliferative growth phase before 9 months of age [3]. This is why we initially discontinued propranolol therapy, something that led to a recurrence. Prolonged growth is observed primarily in IHs with a deep component and segmental morphologic characteristics [3]. Large size and facial location, like in our case, as well as segmental morphology are the most important predictors of poor short-term outcomes, as measured by complication and treatment rates. Segmental hemangiomas are 11 times more likely to experience complications and 8 times more likely to receive treatment than localized hemangiomas, even when controlled for size [4]. 

Physical examination is usually diagnostic in typical cases. Imaging modalities can strengthen the suspected diagnosis in difficult cases. Hemangiomas are usually hypoechoic relative to parotid tissue at ultrasonography and display a variable degree of abnormal flow at Doppler ultrasonography. A color Doppler sonogram typically shows a hypervascular mass with tortuous arterial and venous branches. PHs normally appear isointense with muscle on T1 and hyperintense on T2 sequences, with or without fat saturation. Enlarged and tortuous blood vessels might be seen within and around the tumor. MRI also provides useful information on the size and deep extent of the tumor and its relationship to adjacent structures [5]. 

Although PHs can subside without treatment, their rapid growth can create cosmetic problems along with signs of congestive cardiac failure in cases of significant blood shunting. Moreover, obstruction and/or distortion of nearby structures and ulceration of the mass are potential serious complications. Even though the response rate of PH to medical interventions is similar to that of hemangiomas at other sites, the likelihood of ulceration during the early proliferative phase is much higher in the parotid gland compared to other sites. In a series of 100 consecutive children with PHs, 7% of the patients required tracheostomy and 3% had signs of congestive heart failure [6].

In the past, small well-localized lesions were treated with intralesional corticosteroids, while systemic corticosteroids were indicated for large symptomatic hemangiomas [2, 6, 9]. Long-term systemic corticosteroid therapy is associated with serious side effects such as hypothalamic-pituitary-adrenal suppression, glucose intolerance, weight gain, hypokalemia, sodium and water retention, osteoporosis, and peptic ulcers. Moreover, rebound after administration of corticosteroids is common. Among 22 patients who underwent systemic corticosteroid therapy in the study by Weiss et al., 68% rebounded after cessation of therapy [2]. 

For patients failing corticosteroids or who have a contraindication to corticosteroids, interferon alfa-2a or alfa-2b was considered in the past [2, 3, 6, 9]. However, interferon carries a small but substantial risk of serious neurologic complications, predominantly spastic diplegia, an unacceptable complication [6]. 

Surgery is a potential treatment option for large PHs, provided that it is performed by an experienced team of surgeons. Reinisch et al. retrospectively studied 17 children who underwent surgical resection for PHs. All patients had improvement in facial asymmetry and deformity. However, 2 patients each developed a local hematoma and transient facial nerve palsy, while one patient required a blood transfusion [9]. In recent decades, surgical interventions for PHs have been minimized. None of 20 children treated at the Montreal Children's Hospital required operative management. In that series 100% of the children displayed resolution of their lesions within 2 years of diagnosis with an average follow up of 8.6 ± 5.7 years [10]. 

A group of French investigators were the first to report in 2008 that propranolol inhibits the growth of severe IHs. These investigators proposed three possible mechanisms of action including vasoconstriction, which explains the rapid within days softening of the lesions, decreased expression of the vascular growth factors VEGF and bFGF through the downregulation of their genes by the RAF mitogen-activated protein kinase pathway, and finally activation of apoptosis of capillary endothelial cells [11]. 

Price et al. performed a multicenter retrospective chart review of children with IHs treated with either propranolol or oral corticosteroids. The percentage of clearance was quantified by serial photography and clinical examinations. Overall, 56 of 68 patients (82%) who received propranolol achieved clearance of ≥75% compared with 12 of 42 patients (29%) who received oral corticosteroids, a significant difference. Adverse effects were minimal in the propranolol group, while all 42 patients in the corticosteroids group had ≥1 adverse effects. Moreover, surgical referrals after treatment were required in 12% of the patients in the propranolol group and in 29% in the oral corticosteroids group [12]. In a retrospective study of 39 children with head and neck IHs, propranolol resulted in involution of the lesions at 37 of 39 locations within 2 to 14 days. After successful therapeutic regression, 6 recurrences occurred, but propranolol was effective when retreatment was given [13]. Buckmiller et al. reviewed 32 children treated with propranolol for problematic hemangiomas followed by a blinded analysis of serial photographs taken during therapy. Parental questionnaires were obtained to evaluate perceived therapeutic response and complications. Twenty-seven patients began therapy during the proliferative phase of their lesions, whereas 5 patients began therapy during the involutional phase. Overall, 97% of the patients displayed improvement during propranolol therapy. Ten patients experienced minor side effects to propranolol, including somnolence (27.2%), gastroesophageal reflux (9.1%), RSV exacerbation (4.5%), and rash (4.5%) [14]. De Graaf et al. used oral propranolol for treatment of 28 children (21 girls, mean age 8.8 months) with IHs. All patients had a good response. In 2 patients, systemic corticosteroid therapy was tapered successfully after propranolol was initiated. Side effects that needed intervention and/or close monitoring were not dose dependent and included hypotension (rarely symptomatic), bronchial hyperreactivity, and symptomatic hypoglycemia [15]. Regarding hypoglycemia, Holland et al. described 3 patients with IHs who developed symptomatic hypoglycemia during propranolol therapy [16]. Despite that, propranolol's safety record in infants and children is long-standing and excellent, although questions remain about optimal dosing, time, and way of treatment interruption. Some experts advocate slow tapering of the drug over a 2- to 3-week period, in order to prevent the known problem of rebound tachycardia, although in our experience, this side effect is without clinical consequences in children, in contrast with adults with coronary artery disease [17].

From our paper and the studies quoted above, it appears that oral propranolol at therapeutic doses (2-3 mg/kg/day in divided doses) appears safe for treatment of children with symptomatic hemangiomas, although adverse effects can occur throughout therapy and need vigilance. Thus, propranolol should only be used for complicated hemangiomas, that is, those that due to rapid and prominent growth create functional problems and not for those that create cosmetic problems alone. Despite that, it is our experience and that of others that the relatively uncommon side effects of propranolol are easier to manage compared to the unavoidable and dose-related side effects of systemic corticosteroids.

In conclusion, despite the rushed introduction of propranolol into the clinic without prior head-to-head comparison with the previous medical gold-standard (corticosteroids) [18], we believe that this nonselective b-blocker has revolutionized the treatment of IHs. Propranolol should likely be considered as the first line agent in all infants with severe hemangiomas who require therapy, and who do not have a pulmonary or cardiovascular contraindication to it.
